# Arsenic in Chinese Crayfish: Speciation Analysis, Cooking-Induced Stability, Bioaccessibility, and Dietary Risk Assessment

**DOI:** 10.3390/foods15061068

**Published:** 2026-03-18

**Authors:** Xiaoyi Jiang, Kai Peng, Peng Li

**Affiliations:** College of Food Science and Engineering, Collaborative Innovation Center for Modern Grain Circulation and Safety, Nanjing University of Finance and Economics, Nanjing 210023, China; xiaoyi@nufe.edu.cn (X.J.); 15576389217@163.com (K.P.)

**Keywords:** inorganic arsenic, crayfish, bioaccessibility, risk assessment

## Abstract

Arsenic (As) contamination in aquatic products is a significant public health concern. This study presents a holistic investigation into the speciation, processing stability, bioaccessibility, and health risks of arsenic in crayfish from the Yangtze River basin. The analysis of 60 samples revealed total arsenic (tAs) concentrations ranging from 53.6 to 419.9 μg/kg, with a mean of 109.3 μg/kg. Arsenic occurred predominantly as low-toxicity organic species, with arsenobetaine accounting for 41.3% of tAs on average, while inorganic arsenic (iAs) constituted only 11.6% (mean 12.5 μg/kg). Evaluation of common cooking methods demonstrated that arsenic speciation remained largely stable, with no increase in toxic iAs forms. Notably, boiling in saline water led to significant leaching, reducing iAs content by 28.2%. In vitro gastrointestinal digestion revealed a markedly high bioaccessibility of iAs (81.0–99.3% in the intestinal phase), far exceeding that of tAs (50.4–74.6%). Health risk assessment based on the latest U.S. EPA parameters indicated negligible non-carcinogenic risk across all exposure scenarios. However, the estimated carcinogenic risk for high-intake consumers of high-iAs samples exceeded the acceptable threshold of concern. These findings are expected to provide essential data for understanding the health risks posed by arsenic in crayfish and to support accurate food safety evaluations.

## 1. Introduction

Arsenic (As) is a ubiquitous metalloid in the environment, originating from both natural sources, such as geological weathering and volcanic activity, and anthropogenic activities, including mining, smelting, pesticide application, and fossil fuel combustion [1]. Due to its mobility and bioaccumulative potential, arsenic contamination has emerged as a critical global concern for food safety and public health. The toxicity of arsenic is highly dependent on its chemical species. Inorganic arsenic (iAs), primarily arsenite (As(III)) and arsenate (As(V)), is classified as a Group 1 human carcinogen by the International Agency for Research on Cancer (IARC), associated with an increased risk of various cancers and cardiovascular diseases [2]. In contrast, organic species such as arsenobetaine (AsB), arsenocholine (AsC), monomethylarsonate (MMA), and dimethylarsinate (DMA) are significantly less toxic. Consequently, risk assessment of arsenic in food must evolve from traditional total concentration analysis towards more toxicologically relevant speciation analysis.

Crayfish (*Procambarus clarkii*), a globally consumed freshwater crustacean, has become one of the most representative aquatic consumer products in mainland China. According to the China Crayfish Industry Development Report (2025), the total farming area for crayfish in China reached 2.03 million hectares (30.5 million mu) in 2024, with an annual production of 3.45 million tons in the same year, representing year-on-year increases of 3.39% and 9.07%, respectively [3]. This output accounted for 9.76% of the total freshwater aquaculture production in China, ranking fourth among all the freshwater aquaculture species [3]. As the largest producer and consumer of crayfish, crayfish aquaculture in China is highly concentrated in the middle and lower reaches of the Yangtze River [4]. Aquatic ecosystems serve as important sinks for arsenic, and aquatic products can accumulate large amounts of arsenic through the food chain. Research has been conducted on trace element contamination in crayfish. For instance, Li et al. [5] investigated the enrichment status and health risk assessment of trace elements in Chinese crayfish, pointing out significant differences in spatiotemporal distribution and increased contents of cadmium and arsenic in recent years due to intensive farming practices. Zhang et al. [6] specifically examined the accumulation of toxic elements including lead, mercury, cadmium, arsenic, and copper in red swamp crayfish from Qianjiang City, a leading region for crayfish processing and export, and concluded that the contamination levels did not pose local food safety risks. Li et al. [7] analyzed arsenic species in crayfish from the Yangtze River basin, China, and found that low-toxicity organic arsenic species dominate, with DMA and AsB showing a strong positive correlation. Even so, a critical limitation in existing studies is their predominant focus on the tAs content, which provides insufficient insight into the distribution and risks of iAs and other arsenic species.

Food processing can alter the content and chemical forms of heavy metals [8]. Particularly for aquatic products, thermal treatment during the cooking process can influence both the metal concentrations and speciation. Chi et al. [9] reported that cooking had little effect on the tAs content in shrimp, with boiling and frying increasing the tAs by approximately 3%. Apart from changes in concentration, elemental speciation can also undergo transformation. For example, hexavalent chromium can be reduced to trivalent chromium while interacting with other food components [10]. Likewise, arsenic species are not stable and may also interconvert under different conditions. Under oxidizing conditions, low-toxicity organic arsenic may convert to toxic inorganic forms, whilst reductive conditions can reduce As(V) to the more toxic As(III), thereby increasing potential health risks [11]. Cao et al. [12] observed that the processing of Chinese mitten crabs decreased tAs content and altered the arsenic speciation profiles. In view of the diversity of cooking methods applied to crayfish, whether these processes cause changes in arsenic concentrations or promote the formation of more toxic arsenic species remains largely unexplored.

The total concentration of a contaminant in a food matrix does not directly reflect human exposure. Besides tAs and arsenic speciation, arsenic bioaccessibility is another crucial factor to consider when evaluating dietary exposure. This is defined as the fraction of arsenic that is released from the food matrix into the digestive fluid and becomes potentially available for absorption [13]. It serves as a crucial bridge linking the dietary intake to its potential health risk. In recent years, various in vitro digestion methods have been developed to simulate human gastrointestinal conditions, such as the Physiologically Based Extraction Test (PBET), the in vitro gastrointestinal (IVG) method, and the Simulator of the Human Intestinal Microbial Ecosystem (SHIME) [14,15]. Studies have demonstrated that the bioaccessibility of arsenic varies significantly across digestive stages and food matrices. For instance, Chen et al. [16] reported greater arsenic bioaccessibility in the intestinal phase (47.2–113%) than in the gastric phase (20.1–82.2%) for rice grains by using a modified PBET method. They also observed that Japonica rice exhibited overall lower arsenic bioaccessibility in the intestinal phase compared to Indica rice (71.1% vs. 83.1%). Schmidt et al. [17] investigated the in vitro bioaccessibility of arsenic species in five types of fresh seafood and concluded that the matrix composition has a major influence on the bioavailability of arsenic species. Furthermore, different food processing methods can significantly influence the bioaccessibility of metal elements by altering food composition, texture, and microstructure [18]. However, specific data on the bioaccessibility of iAs in crayfish remains unavailable, representing a significant knowledge gap.

Therefore, we conducted a comprehensive risk assessment of arsenic exposure associated with crayfish consumption, focusing on the abdominal muscle, the primary edible tissue. Specifically, the concentrations and distribution of tAs and six arsenic species (As(III), As(V), MMA, DMA, AsC, AsB) in the abdominal muscle of crayfish from the major aquaculture regions in the middle-lower Yangtze River are determined. The effects of three common cooking methods (frying, boiling, and steaming) on arsenic concentration and speciation stability are then investigated. Subsequently, an in vitro gastrointestinal digestion model is used to evaluate the bioaccessibility of tAs and iAs. Finally, a dietary risk assessment is performed based on the iAs exposure. The findings are expected to provide an essential understanding of the health risks posed by arsenic in crayfish, thereby supporting more accurate food safety evaluations and risk management strategies.

## 2. Materials and Methods

### 2.1. Chemicals and Reagents

Nitric acid (HNO_3_, guaranteed reagent grade) was purchased from Merck Ltd. (Darmstadt, Germany). Hydrogen peroxide (H_2_O_2_, 30%, guaranteed reagent grade) and ammonium dihydrogen phosphate (NH_4_H_2_PO_4_, analytical reagent grade) were obtained from J&K Scientific Ltd. (Beijing, China). Stock standard solutions of total arsenic (1000 mg/L) and six arsenic species (As(III), As(V), MMA, DMA, AsC, and AsB) were acquired from the National Institute of Metrology (Beijing, China). Working standard solutions of lower concentrations were prepared daily via appropriate serial dilution of the stock solutions using ultrapure water (specific resistance ≥ 18.2 MΩ·cm), which was produced by a Millipore water purification system and used throughout all experiments.

For quality control, the certified reference material DORM-4 (fish protein) was obtained from the National Research Council of Canada (Ottawa, Canada). This material was included in each analytical batch to ensure the accuracy and reliability of the arsenic determinations.

### 2.2. Sample Collection and Preparation

**Figure 1:**
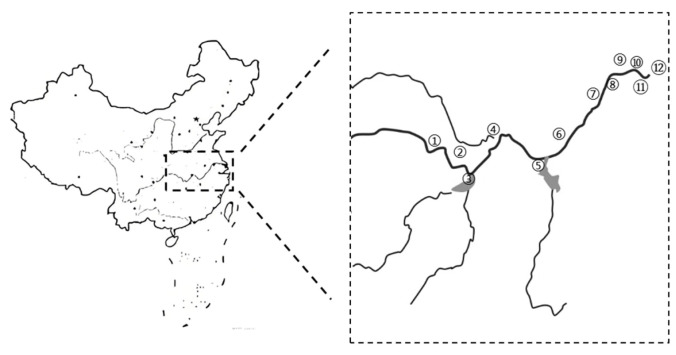
Geographical distribution of crayfish sampling sites in the middle–lower Yangtze River.

In China, five provinces in the middle and lower reaches of the Yangtze River (Hubei, Anhui, Hunan, Jiangsu, and Jiangxi) constitute the core production areas, accounting for 90.45% of the national crayfish output [3]. We selected 12 sampling areas across the five provinces, as well as one major consumer city (Shanghai) to ensure the representativeness of the sampling sites regarding various environmental conditions within each province and the feasibility of sampling and data collection. In each sampling area, five crayfish trading markets were randomly selected for sample collection. A total of 60 crayfish samples were collected from June to August 2022. The sampling locations, as illustrated in the map in Figure 1, included Yichang (①), Qianjiang (②), Yueyang (③), Wuhan (④), Jiujiang (⑤), Anqing (⑥), Wuhu (⑦), Nanjing (⑧), Huaian (⑨), Taizhou (⑩), Wuxi (⑪), and Shanghai (⑫). All collected crayfish were adults. They were transported to the laboratory on the day of purchase, rinsed with purified water to remove attachments, and then dissected. The cephalothorax, telson, shell, and intestinal tract were removed to obtain the abdominal muscle. The abdominal muscle was then minced, thoroughly homogenized, sealed, and stored at −20 °C until analysis for arsenic content.

### 2.3. Determination of Total Arsenic

The determination of tAs was performed following the methodology outlined in our previous study [19]. Briefly, 0.500 g of homogenized muscle sample was weighed into a Teflon digestion vessel in triplicate and subjected to pre-digestion with 5 mL of concentrated HNO_3_ for 1 h at room temperature. Subsequently, 1 mL of H_2_O_2_ was added. The sealed vessel was then placed in a microwave digestion system and subjected to a heating program designed to achieve complete digestion. After cooling to room temperature, the resulting clear digestate was concentrated to approximately 1 mL by evaporation and diluted to a final volume of 10 mL with ultrapure water. The prepared solution was analyzed using an Agilent 7700x ICP-MS (Agilent Technologies, Tokyo, Japan).

The As in the certified reference material DORM-4 was digested and determined in the same manner as the samples. The measured tAs concentration was in good agreement with the certified value of 6.80 ± 0.64 mg/kg, with the relative standard deviation (RSD) ranging from 93.1% to 106.2%.

### 2.4. Extraction and Determination of Arsenic Species

The extraction of arsenic species was performed using a microwave-assisted procedure in accordance with the National Food Safety Standard of China (GB 5009.11-2024) [20], which has been demonstrated to efficiently extract arsenic species while preventing interconversion among them. Briefly, approximately 0.500 g of the homogenized muscle sample was accurately weighed into a Teflon microwave digestion vessel in triplicate. Then, 15 mL of 0.15 M HNO_3_ was added to each vessel. The sealed vessels were placed in a microwave digestion system and subjected to a defined temperature-controlled program (see Table S1). After digestion and cooling, the digestates were centrifuged at 8000 rpm for 15 min. The supernatant was then carefully collected, filtered through a 0.45 μm membrane filter, and stored for subsequent analysis.

The separation and determination of arsenic species were performed using the HPLC-ICP-MS method. Six arsenic species (As(III), As(V), MMA, DMA, AsC, and AsB) were separated on an Agilent 1260 HPLC system (Agilent Technologies, Palo Alto, CA, USA) and quantified by ICP-MS. The eluent from the HPLC was directly introduced into the ICP-MS, and the ICP-MS instrumental parameters were set based on our previous study. Chromatographic separation was achieved using a Hamilton PRP-X100 (250 × 4.1 mm, 10 µm) anion-exchange column (Hamilton, Reno, NV, USA). A gradient elution program was employed with two mobile phases: mobile phase A (ultrapure water) and mobile phase B (25 mM NH_4_H_2_PO_4_, pH adjusted to 8.0). The elution profile was as follows: 0–15 min, A decreasing from 100% to 0%; 15–20 min, A increasing from 0% to 100%; followed by a 5 min post-run equilibration at 100% A.

The limits of detection (LODs) for AsC, AsB, As(III), DMA, MMA, and As(V) were in the range of 1.1~2.0 μg/kg, based on a signal-to-noise ratio of 3:1. Matrix spike recovery experiments were conducted by spiking raw abdominal muscle samples with 20 μg/kg of each arsenic species, with the recoveries ranging from 90.5% to 107.4%. The RSD of repeated measurements (n = 3) was less than 10%. Detailed methodological information is provided in Table S2.

### 2.5. Cooking Process of Crayfish

In this study, 1000 g portions of whole crayfish were subjected to three typical cooking methods: (1) Frying: The samples were deep-fried in soybean oil at 160 °C for 3.0 min. (2) Boiling: The samples were boiled in a 2% (*w*/*v*) NaCl aqueous solution for 10 min. (3) Steaming: The samples were steamed in a steamer for 10 min. After each treatment, the samples were cooled to room temperature. The cooked abdominal muscle was then manually peeled, homogenized, and prepared for subsequent arsenic content analysis. To determine the moisture content of the samples precisely, the abdominal muscle was weighed both before and after being subjected to vacuum freeze-drying, with the moisture content calculated based on the mass loss.

### 2.6. In Vitro Digestion and Bioaccessibility

The in vitro digestion experiment was conducted to simulate the human gastrointestinal environment by adapting the methodology established by Brodkorb et al. [21] with appropriate modifications. The simulated digestive fluids, including simulated salivary fluid (SSF), simulated gastric fluid (SGF), and simulated intestinal fluid (SIF) were prepared according to the described model, which replicates three sequential phases of digestion: oral, gastric, and intestinal. The compositions of the simulated digestive fluids are summarized in Table S3, with the electrolyte stock solutions prepared as specified in Table S4.

The digestion procedure was as follows: First, 5.0 g of steamed and homogenized abdominal muscle sample was mixed with 5 mL of simulated salivary fluid (pH adjusted to 7.0) and incubated in a shaking water bath at 37 °C for 2 min to simulate the oral phase. The resulting oral bolus was then combined with 10 mL of simulated gastric fluid. The pH of the mixture was immediately adjusted to 1.5 using HCl solution, followed by incubation at 37 °C for 60 min with constant shaking to simulate the gastric phase. Upon completion, the gastric digestate was neutralized to pH 7.0 using NaOH solution. Subsequently, 20 mL of simulated intestinal fluid was added, and the mixture was incubated at 37 °C for 120 min with shaking to simulate the intestinal phase. At the end of each respective digestion phase, the digestates were centrifuged. The resulting supernatants were collected and analyzed for arsenic content.

The bioaccessibility of arsenic was calculated as the percentage of arsenic released from the food matrix into the soluble fraction during each digestive phase. It was determined using the following formula:

Bioaccessibility (%) = (C_d_ × V_d_)/(Cs × Ms) × 100

where C_d_ (µg/mL) is the concentration of arsenic in the supernatant after centrifugation of the digestate, V_d_ (mL) is the total volume of the digestate at the end of the respective phase, Cs (µg/g) is the arsenic concentration in the undigested food sample, and Ms (g) is the mass of the food sample subjected to digestion (5.0 g).

### 2.7. Statistical Analysis

Statistical analyses were conducted using SPSS (version 20) (IBM SPSS Inc., Chicago, IL, USA). Data were presented as mean ± SD. One-way ANOVA followed by Dunnett’s post hoc test was used for multiple comparisons with the control group. Statistical significance was set at *p* < 0.05.

## 3. Results and Discussion

### 3.1. Concentration and Speciation of Arsenic in Crayfish Abdominal Muscle

The contents of tAs and its chemical species in the abdominal muscle (primary edible portion) of crayfish from the middle and lower reaches of the Yangtze River were determined. As shown in Table 1, the tAs in the 60 samples varied widely, ranging from 53.6 to 419.9 μg/kg, with a mean value of 109.3 μg/kg and a median of 91.1 μg/kg. This distribution indicated a positive skew, with a few samples exhibiting notably high arsenic values. The highest arsenic content was observed in a sample sourced from Jiangsu Province, which might be associated with local industrial activities. Notably, the mean tAs concentration in the edible portion of crayfish (109.3 μg/kg) was higher than that reported for many other food products, such as rice, milk, and various aquatic products from several regions [22,23].

The arsenic speciation analysis, however, revealed a distribution pattern dominated by low-toxicity organic species. Specifically, AsB, DMA, and AsC were the three most abundant species, with their combined mean concentrations accounting for 78.0% of the total measured arsenic species. In contrast, the toxic iAs was present at substantially lower levels, with a mean concentration of 12.5 μg/kg, representing only 11.6% of the mean tAs. Furthermore, the 90th percentile value for iAs was 16.4 μg/kg, indicating that even in samples with higher overall arsenic burden, the absolute concentration of iAs remained rather low. The maximum detected iAs concentration (80.7 μg/kg) was greatly below both the European Union maximum level for iAs in crustaceans (0.1 mg/kg) and the Chinese national standard limit for iAs in aquatic products (0.5 mg/kg) [24,25].

The observed speciation profile, characterized by the predominance of low-toxicity AsB (mean proportion of 41.3%) and a relatively low proportion of iAs (11.6%), aligns with the general metabolic and detoxification pathways of arsenic in most aquatic organisms. Aquatic species, particularly crustaceans and fish, typically metabolize absorbed iAs through a series of biomethylation steps, producing MMA and DMA with lower toxicity, which are ultimately converted into the virtually non-toxic AsB that can be stably stored in tissues such as muscle [26]. The observed dominance of AsB in this study suggests the crayfish possess efficient biomethylation and conversion capacity to detoxify and transform iAs into organic storage forms, despite inhabiting a freshwater environment where dissolved arsenic is primarily inorganic. In addition, the mass balance calculation was performed by comparing the sum of individual arsenic species (ΣAs species) with the measured tAs concentration. The results showed that ΣAs species accounted for 92.7% of the tAs on average. Although this mass balance falls within the acceptable range for arsenic speciation analysis, the incomplete recovery (approximately 7.3% unaccounted for) suggests the possible presence of other arsenic species that were not detected or quantified by the current analytical method.

### 3.2. Effect of Cooking Process on Arsenic and Its Speciation

The impact of common domestic cooking methods (frying, boiling, and steaming) on the levels and speciation of arsenic in crayfish abdominal muscle was evaluated. All thermal treatments caused a significant reduction in moisture content, decreasing from 79.7% in raw meat to 59.1%, 57.3%, and 63.1% in fried, boiled, and steamed samples, respectively. All concentrations were expressed on a dry-weight basis to eliminate the influence of moisture loss and compare the intrinsic arsenic content.

**Figure 2:**
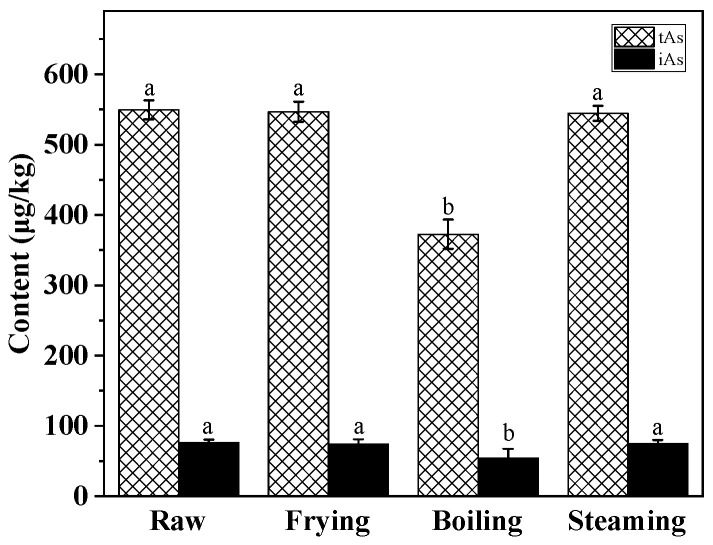
Contents of tAs and iAs under different cooking processes (dry weight). The error bars represent the standard deviation (n = 5). Different letters within the tAs and iAs groups indicate significant differences among samples at the *p* < 0.05 level.

The results revealed distinct effects among the processing methods. First, frying and steaming did not induce significant changes in the concentrations of either tAs or iAs (Figure 2). In contrast, boiling in saline water led to a significant reduction (*p* < 0.05) in both tAs and iAs, by 32.2% and 28.2%, respectively. This finding aligns with the phenomenon observed by Schmidt et al. [27], where analyte transference to the cooking medium was identified as a key factor altering arsenic concentration. By analyzing the boiling water, it was confirmed that the decrease in tAs and iAs can be primarily attributed to the leaching of water-soluble arsenic species into the aqueous boiling medium. Conversely, the stability of arsenic concentrations during frying is likely due to the low permeability of the oil-based medium. The rapid protein coagulation induced by high heat forms a physical barrier that traps analytes within the matrix [28]. For steaming, although it involves an aqueous environment, the absence of a significant reduction may result from the lack of a concentration gradient. Unlike boiling, where continuous immersion facilitates leaching into the cooking water, steam condensation on the sample surface appears insufficient to drive the extraction of arsenic. Speciation analysis further revealed that the decrease in iAs in the boiling sample was primarily attributed to a decrease in As(V). This phenomenon may be attributed to the disruption of electrostatic interactions that originally exists between As(V) and the protein matrix of the meat, and the high ionic strength of the saline cooking medium results in the leaching of As(V) into the cooking water [29,30]. Unlike As(V), As(III) in food is primarily bound to proteins via covalent interactions with sulfhydryl groups [31]. These covalent bonds remain stable under boiling and high-salt conditions, thereby limiting the release of As(III) into the cooking medium.

Considering that the reductive conditions can potentially convert As(V) to the more toxic As(III), a supplementary experiment was conducted by adding common antioxidant condiments (ginger and garlic) during frying. No such conversion was detected, indicating that these reductive transformations did not occur under the tested cooking conditions. Although some studies have reported the conversion of AsB to more toxic species in certain cooked seafood products [32], our findings demonstrate that arsenic speciation in crayfish abdominal muscle remains largely stable during common cooking processes. Importantly, none of the investigated methods promoted the formation or accumulation of toxic iAs species.

### 3.3. Bioaccessibility of Total Arsenic and Inorganic Arsenic

**Figure 3:**
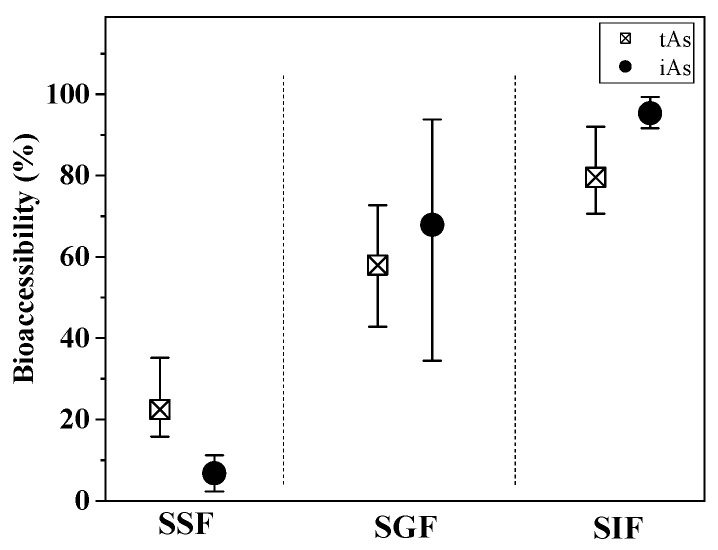
Bioaccessibility of tAs and iAs in SSF, SGF, and SIF across 10 different crayfish samples. The icons represent the mean values; the upper and lower bars indicate the minimum and maximum values.

The bioaccessibility of tAs and iAs in crayfish abdominal muscle during simulated oral, gastric, and intestinal digestion was investigated. As shown in Figure 3, the results revealed clear differences in the release patterns of tAs and iAs as digestion progressed. The released tAs exhibited a progressive pattern, with low bioaccessibility in the oral phase (11.3–25.1%), rising in the gastric phase (30.6–51.9%), and reaching the highest levels in the intestinal phase (50.4–74.6%). This stepwise release aligns with the sequential digestion of protein-based foods, where the acidic environment in the stomach and the enzymatic digestion in the intestine successively break down muscle tissue and protein–arsenic complexes, thereby liberating more bound arsenic.

In contrast, the bioaccessibility of iAs followed a markedly different release behavior. Only minor release was observed in the oral phase (0–11.2%), followed by a rapid increase in the gastric phase (34.4–93.8%) and nearly complete release (81.0–99.3%) in intestinal phase. By the end of digestion, almost all the iAs had been released, whereas a substantial fraction of tAs remained bound within the protein matrix, highlighting much higher solubility and mobility of iAs than tAs under gastrointestinal conditions. This also strongly suggests that the less toxic organic forms of arsenic are more resistant to gastrointestinal digestive breakdown and release compared with toxic iAs.

The bioaccessibility of elements in food is strongly influenced by the food matrix. A study on arsenic bioaccessibility in fishes, shellfishes, and seaweeds from Chinese markets reported considerable variation among seafood types, with higher tAs bioaccessibility observed in fishes (gastric: 68.6%; intestinal: 81.9%) compared to shellfishes (gastric: 40.9%; intestinal: 52.5%) and seaweeds (gastric: 31.0%; intestinal: 53.6%) [33]. Other studies have reported iAs bioaccessibility values as high as 92% and 94% in various shellfishes and fish [34]. In the present study, the mean bioaccessibility values for tAs and iAs were 82.0% and 95.3%, respectively, which are generally consistent with those reported for typical seafood products. However, it should also be acknowledged that in vitro digestion models cannot fully replicate the complex physiological environment of the human body. Factors such as the dynamic secretion of digestive enzymes, gastrointestinal peristalsis, the intestinal absorption barrier, and inter-individual physiological variations are not adequately accounted for [35]. As a result, bioaccessibility measured in vitro may deviate from actual in vivo bioavailability, potentially leading to either an overestimation or underestimation of the true absorption levels of elements.

### 3.4. Health Risk Assessment

Based on the aforementioned research findings, the potential health risks associated with arsenic exposure from crayfish consumption were assessed. Since the cooking processes did not cause an increase in iAs content and the bioaccessibility analysis indicated a high gastrointestinal release rate (over 90%) of iAs, the risk assessment was conducted directly based on the iAs concentration in the abdominal muscle of crayfish.

#### 3.4.1. Estimated Daily Intake (EDI)

The Estimated Daily Intake (EDI) was calculated to evaluate the dietary exposure level of iAs using the following formula:

EDI (mg/kg bw/day) = (C_i_ × D_i_)/BW

where C_i_ is the iAs concentration in abdominal muscle (mg/kg), D_i_ is the daily consumption (kg/day), and BW is the body weight (kg). For samples with iAs below the LOD, 1/2 LOD was assigned to C_i_. The daily consumption of crayfish was based on a published and widely recognized survey [36], which provided detailed quantitative data on crayfish consumption patterns among local residents, including both mean (10.1 g/day) and high (27.6 g/day) consumption values. As 92% of the survey respondents were adults, a consumer body weight of 60 kg was adopted for the exposure assessment. In order to comprehensively evaluate the risk, four exposure scenarios were established by combining the mean (0.0125 mg/kg) and maximum (0.0807 mg/kg) iAs concentration with the mean (10.1 g/day) and maximum (27.6 g/day) daily consumption. The calculation results are presented in Table 2.

The calculated EDI values ranged from 2.1 × 10^−6^ to 3.7 × 10^−5^ mg/kg bw/day, which were significantly lower than the previously reported EDIs of iAs from other aquatic products such as crab, fish, and shrimp (from 0.1 × 10^−4^ to 3.9 × 10^−4^ mg/kg bw/day) [25].

#### 3.4.2. Target Hazard Quotient (THQ)

The Target Hazard Quotient (THQ) was calculated to assess the chronic non-carcinogenic risk from long-term exposure as follows:

THQ = EDI/RfD

where RfD is the oral reference dose for iAs. This study adopted the latest value of 0.00006 mg/kg/day, from the U.S. Environmental Protection Agency’s (U.S. EPA) Integrated Risk Information System (IRIS) updated in 2025 [37]. According to international standards, a THQ < 1 indicates an acceptable non-carcinogenic risk, while a THQ > 1 suggests a potential risk that may require attention [38]. As shown in Table 2, the THQ values across all scenarios ranged from 0.035 to 0.619, all well below the safety threshold of 1. This indicates that the chronic non-carcinogenic risk from iAs intake through crayfish consumption is low and acceptable, even for consumers with the highest intake of samples containing the highest iAs content.

#### 3.4.3. Target Cancer Risk (TCR)

The Target Cancer Risk (TCR) was used to evaluate the carcinogenic risk of iAs. It was calculated using the Oral Slope Factor (OSF) specific to cancers such as bladder and lung cancer as follows:

TCR = EDI × OSF

where the OSF value of 32 (mg·kg^−1^ bw·day^−1^)^−1^ from the latest assessment released by the U.S. EPA IRIS in 2025 was applied [37]. The OSF is a composite slope factor based on the risks of bladder and lung cancer. According to the established international criteria, a TCR < 10^−6^ is considered a negligible risk, values between 10^−6^ and 10^−4^ indicate a potential manageable risk, and TCR > 10^−4^ warrants concern [39].

As shown in Table 2, TCR values under average exposure scenarios all fell within the “potential manageable risk” range (10^−6^ to 10^−4^). However, under the most conservative high-exposure scenario (high consumption and high iAs content), the TCR value reaches 1.188 × 10^−3^, exceeding the commonly accepted concern threshold of 10^−4^. While the carcinogenic risk remains within a potentially acceptable range under average consumption patterns, the assessment based on the latest and more stringent OSF parameter highlights a potential lifetime cancer risk for high-exposure subpopulations (e.g., frequent consumers with high intake). These findings underscore the importance of targeted dietary guidance for high-consumption groups and demonstrate the value of applying updated, health-protective toxicological parameters in refined risk assessment.

## 4. Conclusions

This study demonstrates that arsenic in commercially sourced crayfish from the Yangtze River basin primarily exists as low-toxicity organic forms, and cooking processes do not increase toxic iAs. The key finding is the near-complete bioaccessibility of iAs during gastrointestinal digestion, in contrast to the partial release of tAs, highlighting a substantial difference between total content and actual exposure potential. Risk assessment indicated negligible chronic health risk for average consumers, but suggested a potential concern regarding lifetime cancer risk for a small subgroup with extremely high intake. These results emphasize that arsenic speciation and bioaccessibility, rather than total arsenic concentration alone, are critical determinants for accurate dietary risk evaluation of crayfish.

## Figures and Tables

**Table 1:** Concentration and proportion of arsenic species in crayfish abdominal muscle (wet weight).

Arsenic Species	Minimum (μg/kg)	Maximum (μg/kg)	Mean (μg/kg)	Median (μg/kg)	90th Percentile (μg/kg)	Mean Proportion (%)
tAs	53.6	419.9	109.3	91.1	160.8	-
AsC	ND *	276.7	16.6	2.5	11.9	15.6
AsB	18.9	106.6	44.2	37.9	68.4	41.3
As(III)	ND	55.6	9.0	5.6	13.6	8.4
DMA	11.1	59.2	22.8	18.1	36.1	21.3
MMA	ND	10.7	5.8	5.5	8.8	5.4
As(V)	ND	25.1	3.4	2.3	5.5	3.2
iAs	ND	80.7	12.5	8.5	16.4	11.6

* ND means not detected.

**Table 2:** Exposure and risk characterization of inorganic arsenic under different scenarios.

iAs in Sample (mg/kg)	EDI (mg/kg bw/Day)		THQ		TCR	
	Average Intake ^a^	High Intake ^b^	Average Intake	High Intake	Average Intake	High Intake
0.0125 (mean)	2.104 × 10^−6^	5.750 × 10^−6^	0.035	0.096	6.733 × 10^−5^	1.841 × 10^−4^
0.0807 (maximum)	1.358 × 10^−5^	3.712 × 10^−5^	0.226	0.619	4.347 × 10^−4^	1.188 × 10^−3^

^a^ 10.1 g abdominal muscle per day. ^b^ 27.6 g abdominal muscle per day.

## Data Availability

The original contributions presented in the study are included in the article; further inquiries can be directed to the corresponding author.

## Supplementary Materials

The following supporting information can be downloaded at https://www.mdpi.com/article/10.3390/foods15061068/s1: Table S1: Microwave-assisted extraction program. Table S2. Methodological parameters for the analysis of arsenic species. Table S3: Configuration table of simulated digestive solution. Table S4: Compositions of electrolyte stock solutions for simulated digestive fluids.
