# Supplementary material for: Arsenic in Chinese Crayfish: Speciation Analysis, Cooking-Induced Stability, Bioaccessibility, and Dietary Risk Assessment
Source: Foods. 2026 Mar 18;15(6):1068. doi: 10.3390/foods15061068 (PMC13025128; doi:10.3390/foods15061068)
Supplement: Supplementary file 1 [file foods-15-01068-s001.zip › foods-4179196-supplementary.pdf]

Supplementary Material for the article:

Arsenic in Chinese crayfish: speciation analysis, cooking-induced stability, bioaccessibility, and dietary risk assessment

Xiaoyi Jiang, Kai Peng and Peng Li*

College of Food Science and Engineering, Collaborative Innovation Center for Modern Grain Circulation and Safety, Nanjing University of Finance and Economics, Nanjing 210023, China; xiaoyi@nufe.edu.cn (X.J.); 363124282@qq.com (P.K.)
* Correspondence: lipengim@126.com; Tel.: +86-025-86718515

Table S1. Microwave-assisted extraction program.

Step	Power (W)	Set Temperature (°C)	Hold Time (min)
1	1600	70	10
2	1600	90	10
3	1600	110	15

Table S2. Methodological parameters for the analysis of arsenic species.

Arsenic Species	Calibration curve (R^2)	LOD (μg/kg)	Recovery (%)	RSD (%, n=3)
AsC	0.2~100 μg/L (> 0.999)	1.2	90.5	7.6
AsB	0.2~100 μg/L (> 0.999)	2.0	96.2	5.3
As(III)	0.2~100 μg/L (> 0.999)	1.7	95.4	2.4
DMA	0.2~100 μg/L (> 0.999)	1.1	102.8	3.1
MMA	0.2~100 μg/L (> 0.999)	1.5	107.4	2.7
As(V)	0.2~100 μg/L (> 0.999)	1.8	91.3	4.3

Table S3. Configuration table of simulated digestive solution.

Digestion phase	Oral (SSF)	Gastric (SGF)		Intestinal (SIF)	
Food or digesta	5 g of sample	10 mL from oral phase		20 mL from gastric phase	
Electrolyte stock solutions	5 mL	10 mL		10 mL	
0.3 M $CaCl_2(H_2O)_2$	0.025 mL	0.005 mL		0.04 mL	
Enzyme	Salivary amylase (1000 U/mg), 0.75 mg	Pepsin (2500 U/mg), 16	Gastric lipase (30	Trypsin (2500 U/mg),	Bile salts (Bile acid

		mg	U/mg), 40 mg	1.6 mg	content ≥ 75%), 218 mg
pH	7.0	1.5		7.0	
Final volume	10 mL	20 mL		40 mL	

Table S4. Compositions of electrolyte stock solutions for simulated digestive fluids.

Reagent	SSF (pH 7) (mmol/L)	SGF (pH 1.5) (mmol/L)	SIF (pH 7) (mmol/L)
KCl	15.1	6.9	6.8
KH_2PO_4	3.7	0.9	0.8
$NaHCO_3$	13.6	25.0	85.0
NaCl	1.08	47.20	38.40
$MgCl_2 \cdot 6H_2O$	0.15	0.12	0.33
$(NH_4)_2CO_3$	0.06	0.50	—
